# Use of Indigenous-Based Methodologies to Enhance the Understanding of Local Context in Ugandan Communities: Protocol for a Mixed Methods Study

**DOI:** 10.2196/75136

**Published:** 2025-10-16

**Authors:** Sahr Wali, Jeremy I Schwartz, Justice Seidel, Jenipher Kamarembo, Jenifer Atala, Ann R Akiteng, Martha Nabadda, Cinderella N Muhangi, Angela Mashford-Pringle, Heather Ross, Joseph Cafazzo, Isaac Ssinabulya

**Affiliations:** 1 Dalla Lana School of Public Health Institute of Health Policy, Management and Evaluation University of Toronto Toronto, ON Canada; 2 Centre for Digital Therapeutics University Health Network Toronto, ON Canada; 3 Ted Rogers Centre for Heart Research University Health Network Toronto, ON Canada; 4 Uganda Initiative for Integrated Management of Non-Communicable Diseases Kampala Uganda; 5 Section of General Internal Medicine Yale School of Medicine Yale University New Haven, CT United States; 6 NOSM University Thunder Bay Canada; 7 Uganda Heart Institute Mulago Hospital Kampala Uganda; 8 Lira Regional Referral Hospital Lira Uganda; 9 Waakebiness-Bryce Institute for Indigenous Health Dalla Lana School of Public Health University of Toronto Toronto, ON Canada; 10 Institute of Medical Sciences Faculty of Medicine University of Toronto Toronto, ON Canada; 11 Department of Computer Science University of Toronto Toronto, ON Canada

**Keywords:** community-based research, digital health, heart failure, participatory research, user-centered design

## Abstract

**Background:**

With many socially disadvantaged populations experiencing a higher level of illness than the general population, health research has begun to recognize the impact of social determinants on health outcomes. Community-based research has increasingly been used to understand the complexities of the local context. However, given the number of interdependent factors influencing individual well-being, no single methodology can explore this level of complexity alone. To put context into perspective, research processes need to shift from the sole use of Western methodologies and, instead, incorporate collaborative methods from nontraditional research. Specifically, Indigenous methodologies have been developed to better understand the complexity of context within multiple worldviews, but current studies have failed to apply these approaches within other cultural settings.

**Objective:**

This mixed methods study will use Western and Indigenous methodologies to adapt a digital health program for remote communities in Uganda.

**Methods:**

Using the principles of community-based research and user-centered design, a 4-phase mixed methods study will be conducted. The Indigenous method of 2-eyed seeing will be used to promote a reflexive engagement strategy throughout all study phases. Phase 1 will focus on partnership building to codevelop the project priorities and study design. Phase 2 will involve a needs assessment to elicit a context-focused understanding of the local clinic and community environment. Phase 3 will involve a series of system adaptations to co-design the program. Phase 4 will consist of a community-based field study to evaluate the usability and cultural relevance of the adapted program.

**Results:**

This study was approved by the Makerere University School of Medicine Research and Ethics Committee (Mak-SOMREC-2021-63) and the University Health Network Research Ethics Board (20-6022). This protocol provides a novel strategy leveraging a range of community-based methods to ensure that the contextual significance of each community’s challenges is reflected in the design of the Medly Uganda program. Partnership building was initiated in June 2019, and the first stage of data collection in phase 2 began in January 2021. At the time of manuscript submission, phases 1 to 3 have been completed. Phase 4 data analysis is ongoing and expected to be completed in October 2025.

**Conclusions:**

Integrating the community’s local knowledge into the design of the Medly Uganda program will lead to the development of meaningful interventions that improve health outcomes.

**International Registered Report Identifier (IRRID):**

DERR1-10.2196/75136

## Introduction

Over the past few decades, health research has begun to recognize the influential impact of various social determinants on individual health and well-being [1,2]. Specific contextual environments have been associated with poorer population health outcomes simply due to the conditions individuals are born into [1,3]. With a variance in social, political, and environmental settings, a community’s well-being is often shaped through its accessibility to basic human necessities such as housing, food, water, education, safety, and employment [2-4]. Thus, many underserved, low-income, and socially disadvantaged populations face a significantly higher level of illness due to complex factors related to poverty, social stratification, and overall disparities in their social determinants of health [1,2,4]. To better support the unique needs of these communities, understanding the state of their contextual environment and the underlying contributors influencing their health outcomes is pivotal.

With several evolving and interdependent factors contributing to the disparities in a population’s social determinants of health, many studies recognize that no single methodology can explore or understand this level of complexity alone [3,5]. Instead, to improve a health service or develop a care program in reflection of these contextual factors, using a combination of collaborative approaches will lead to the development of a more effective research strategy. Community-based participatory research (CBPR) is being increasingly used to support the production of knowledge in a manner that places local voices at the center of a partnership to promote meaningful change [5-7]. The lived experiences and unique perspectives provided by community members allow for a deeper sense of context to be explored, but it is important to recognize that, as each individual comes from certain positionalities, they express different worldviews [3,5,8]. The definition of health and healing can vary within different cultural settings, whereby the reality of specific circumstances can even be experienced and shared by individuals in different ways [5]. Thus, to allow for multiple perspectives to be better recognized for their own value, there is a need for research processes to shift from the predominant focus on Western biomedicine and, instead, recognize the value and strengths of different cultural worldviews.

Compared to Western notions of dichotomous thinking and individualism, many Indigenous knowledge frameworks embrace the process of reflexivity, collaboration, and interconnectedness to better understand multiple worldviews [9,10]. These frameworks recognize the importance of reflecting on one’s positionality and personal motivations to conduct research, to the extent that the intention and process of data collection are held at equal importance to that of the knowledge acquired [9,11]. Through this paradigm, CBPR is able to set the foundation for understanding community context, but Indigenous methodologies are able to serve as the vehicle to translate the principles of partnership and self-reflection into practice [5,9]. Given that the term *Indigenous* refers to any population or group of people originating from a specific region before colonization and modern geographical borders were defined, we recognize that different Indigenous methodologies have been developed in reflection of a community’s cultural values [10]. Notably, across Canada and Australia, Indigenous researchers or knowledge keepers have conducted decades of extensive evaluative research to develop frameworks that uphold the strengths of cultural knowledge within the process of traditional research practice [5,10,11]. While Indigenous and non-Indigenous communities outside Canada and Australia may face varying challenges within their local environment, the integrative nature of these methods allows for the exploration of a deeper understanding of community context [9,11]. Other participatory methods may involve community members in data collection, but Indigenous knowledge frameworks provide a deeper outlook on the knowledge gathered and how it relates to different dimensions of well-being beyond just one’s physical health. Despite these benefits, current studies have failed to apply these methods within other cultural settings, and the course of pilot projects failing to meet local community needs continues [9,11]. To address this gap and better understand the contextual challenges faced by underserved communities, this protocol paper outlines a mixed methods study using both Western and Indigenous methodologies to adapt a digital health program for remote communities in Uganda. We anticipate that the use of community knowledge and clinical expertise will lead to the development of a more impactful and culturally relevant digital health program that will improve care outcomes.

## Methods

### Medly Uganda: A Digital Health Program for Remote Heart Failure Care

In Uganda, a substantial level of health disparities has been reported as many communities currently live below the poverty line [4]. With the burden of noncommunicable diseases continuing to grow, the interplay between poverty and health care access has been found to be one of the largest contributors to poorer health outcomes [4,12]. Specifically, community risk factors related to food insecurity, unemployment, and poorly resourced health care systems have led many patients living with cardiovascular diseases such as heart failure (HF) to present advanced stages of the disease early in their diagnosis [12-14]. Despite the severity of the disease, HF is largely treatable or controllable if patients are given the tools to support their ability to self-care [15-17]. However, with the lack of health resources available in Uganda, there is a need for affordable strategies to be introduced for the benefits of self-care to be realized [14,18].

Given the widespread penetration of mobile phones, digital health has emerged as a unique opportunity to assist populations with limited resources [19,20]. By leveraging the use of ubiquitous technologies such as mobile phones, digital health interventions are able to provide evidence-based, individualized care support (ie, education, lifestyle reminders, and decision support) directly to patients [19-21]. The Uganda National Panel Survey of 2019 to 2020 estimated that approximately 74% of all households owned a mobile phone; thus, digital health can serve as a low-cost option to provide clinical expertise and remote care support [22-24]. Recognizing the benefits of digital health, in 2016, the Uganda Heart Institute (UHI), the University Health Network (UHN), and Yale University collaborated to adapt a mobile phone–based app for HF self-care developed and tested in Canada for the Ugandan setting [25]. To guide the adaptation of the mobile phone–based program, known as Medly Uganda, a mixed methods study including 101 patient surveys and a series of in-depth patient, caregiver, and clinician interviews was conducted [25]. Building on this work, a prospective cohort study was recently conducted at the UHI in Kampala, Uganda, to evaluate the effectiveness of the Medly Uganda program on patient outcomes (ClinicalTrials.gov identifier NCT04426630) [26]. By generating tailored self-care advice based on patient-centered symptoms and providing remote nursing support, the Medly Uganda program was able to demonstrate improvements in patient self-care and health-related quality of life with respect to HF [26].

### Research Aim

Despite the positive findings of the aforementioned Medly Uganda pilot study, it is important to recognize that patients in this study had the financial resources to travel to the UHI or the benefit of residing in the capital city, where specialized expertise and clinical resources are more readily available [12,13]. With approximately 84% of Ugandans residing in rural communities, rural access to health care remains a challenge. Urban bias has led many of the poorest communities with the highest health care needs to receive the least care support [12,13]. Given these conditions, the Medly Uganda program has the potential to provide greater benefits for communities residing in rural areas with limited access to care, but the unique needs of each community would need to be reflected in the program’s design. To facilitate this aim, this mixed methods study will work in partnership with remote clinics in Uganda to identify the cultural and service-level requirements to adapt the Medly Uganda program.

### Study Design

The principles of CBPR and user-centered design (UCD) will be used to conduct a 4-phase mixed methods study for the remote clinic adaptation of the Medly Uganda program [22,27]. In many digital health–focused studies, researchers have often arrived in communities with pre-established research agendas without understanding the drivers or barriers leading to their health inequities [24,27]. To better understand the local context and empower the local Ugandan voice, a series of Indigenous-based methodologies originating in Canada and Australia will also be used to promote a reflexive community engagement strategy throughout all stages of the study. Specifically, the “Nothing about us without us” tenet, focused on partnership, mutual learning, and informed co-design, will be used as the theoretical paradigm for this study [28].

To initiate the community-based adaptation of the Medly Uganda program, phase 1 of this study will be centered on partnership building to codevelop the project priorities, study design, and methods for data collection and analysis. Phase 2 involves the conduct of a needs assessment with patients and health care providers to elicit a context-focused understanding of the local clinic and community environment, as well as the cultural and service-level needs to adapt the program. On the basis of the feedback from phases 1 and 2, phase 3 will involve an iterative series of system adaptations to co-design the Medly Uganda program to meet the local needs highlighted in phase 1. Finally, to evaluate the changes to the adapted system, phase 4 will consist of a community-based field study to evaluate the usability and cultural relevance of the adapted tool.

### Phase 1: Partnership Building

#### Overview

As part of the Rheumatic Heart Disease Research Collaborative, the UHI currently works with 4 clinics in the central, western, and northern regions of Uganda (ie, Lubowa, Mbarara, Gulu, and Lira) [29]. On the basis of guidance from UHI stakeholders in Kampala, community outreach to the northern clinics in Lira and Gulu will first be initiated as a greater need for care support was expressed by their local teams. To ensure that respectful engagement strategies are used throughout the partnership-building process, the Intervention and Research Readiness Engagement and Assessment of Community Health Care (I-RREACH) tool will be used [22]. The I-RREACH tool, developed with Indigenous communities in Canada, is a community-based engagement and assessment resource comprising (1) Indigenous participatory consensus cycles, (2) a community profile tool, (3) a key informant interview guide, (4) a focus group guide, and (5) a participant evaluation survey to support the implementation of interventions in low-resource environments [22]. Various components of the I-RREACH tool will be used throughout this study to ensure that the cultural and lived experiences of the community are guiding the adaptation of the Medly Uganda program.

To initiate the partnership-building phase, a series of engagement sessions with each clinic’s (ie, in Gulu and Lira) key clinical (ie, nurses, administrators, and clinicians) and community (ie, village health teams [VHTs], parish coordinators, and community leaders) stakeholders will be conducted using the I-RREACH tool’s participatory consensus cycles [22]. Each stakeholder will be asked to participate in a minimum of 3 engagement sessions to help codevelop the project priorities and overall study approach. This process will consist of a negotiation built on trust, honesty, and reciprocity between stakeholders. To identify the participants for the consensus cycles, this study will work collaboratively with the lead research nurse from each clinic to leverage their guidance on the appropriate clinical and community representatives.

#### Cycle 1: Initiation

The first engagement session will serve as an introductory discussion to explore each community’s current care priorities, as well as the local perspective regarding the proposed research approach. The study team will begin the engagement session by presenting their skills and interests regarding collaboration. The stakeholders will be informed that, despite the study team’s given skills and interests, the study objectives and program adaptations will be determined by what the clinic and community set as a priority. A guided discussion with the clinic and community stakeholders will then commence to broadly explore and identify the scope of the challenges present. To elicit a rich dialogue based on previous research evidence, we have previously completed two scoping reviews investigating (1) the use of digital interventions in low-resource settings and (2) the various community engagement strategies to co-design health interventions [10,24]. The findings of these reviews will be discussed in relation to the stakeholders’ lived experience to ensure that the topics are explored in a manner reflecting the local community conditions. At the end of the engagement session, participants will complete the adapted I-RREACH clinic profile community demographic survey. The study team will also summarize the dialogue exchanged for the stakeholders to further review, discuss, and modify.

#### Cycle 2: Validation

The second engagement session will use the process of member checking, also known as respondent validation, to confirm each stakeholder’s perspective regarding the proposed research approach. To facilitate this aim, a summary of the program objectives based on the feedback from cycle 1 will be provided to all stakeholders during an in-person community site visit. This will allow the study team to formally collect each stakeholder’s views regarding whether the program objectives reflect their own beliefs and priorities. If a mutual agreement regarding the study objectives is not achieved by the end of cycle 2, additional engagement sessions will be held for further collaborative discussion.

#### Cycle 3: Finalization

The third engagement session will serve as the final member checking to validate the study’s objectives and overall research approach. A summarized report of the previous engagement sessions will be provided to each stakeholder, where they will subsequently be asked to provide written feedback on their agreement with the outlined study objectives. Stakeholders will also be asked to complete a questionnaire to confirm their level of involvement within the study (ie, data collection, data analysis, knowledge translation, and advisory), as well as to provide their suggestions for some of the practical aspects of the study regarding the recruitment strategy, interview location, study start date, and community outreach.

### Phase 2: Engagement and Needs Assessment

#### Study Procedures

Following the approval of the study approach and project objectives by the clinic and community stakeholders, the study team will conduct an iterative needs assessment with a convenience sample of patients with HF, clinicians, and VHTs at each clinic. On the basis of community partner guidance regarding patient availability and staff workflows, we aim to recruit a total of 15 patients, 10 clinicians, and 5 VHTs to participate from each of the clinics in Lira and Gulu. At the beginning of the session, participants will be given an overview of the study objectives, as well as the opportunity to ask questions before providing written informed consent. With the support of each clinic’s lead nurse, we will provide participants with the option to conduct the session in English or their local language (ie, Acholi, Luo, Runyankole, and Langi). Once written informed consent is obtained, a series of yarning-style semistructured interviews will be conducted to discuss each participant’s perceptions of the contextual influencers affecting HF outcomes and the cultural and service-level needs to adapt the Medly Uganda program. The process of yarning allows interview discussion guides to move away from directed questions and more toward relationship building through the exchange of real stories. By using the Indigenous method of yarning, participants will be able to share their stories and lived experiences in a more conversational approach that embraces different cultural values [30,31]. This study will work with clinic partners to codevelop the interview guide and integrate the yarning style based on relationship-building strategies used within Ugandan culture. For example, the use of the radio for information gathering is often adopted, as well as strong family ties for caregiver support. Participants will also be asked to complete a postinterview evaluation survey and a demographic survey consisting of questions regarding their lifestyle, access to care, community support, medication availability, cell phone access, and disease conditions. Using the I-RREACH tool’s interview guide, participant evaluation survey, and community profile sheet, all data collection instruments will be codeveloped with the community’s core stakeholders to ensure that the questions are both relevant and appropriate.

#### Recruitment

The study team will work in partnership with the local clinic and community stakeholders to initiate a recruitment strategy at each site. This may include the use of posters, community outreach initiatives, clinic referral, or self-referral. With support and leadership from the local clinic team, we will invite clinicians and VHTs for participation through both verbal and written (ie, email and posters) invitations. Individuals who express interest in joining the study will be connected with a member of the study team through a phone call. The study team will then provide the clinician or VHT with information regarding the study procedures and will offer them the opportunity to schedule a time for an in-person interview. To optimize inclusivity and the diversity of the participants, we will aim to recruit individuals of varying genders, ages, and socioeconomic statuses.

Patient recruitment will follow a similar procedure, where the local clinic and community leadership will help spearhead the recruitment of eligible patients from each site. Eligible patients interested in the study will be connected with a member of the study team to review the various study procedures, and they will then be asked whether they would like to participate in the interview or survey. Once written informed consent is obtained from each patient, interviews will be conducted in a private room at each clinic. All participants who complete phase 1 will be asked to participate in the next study phases, but this will not be a requirement.

The participant inclusion criteria are presented in Textbox 1.

**Inclusion criteria.**

**Health care providers**

- Physicians or nurses working at a Lira or Gulu clinic

**Village health teams (VHTs)**

- VHTs or community health workers providing care support within Lira or Gulu communities

**Patients**

- Aged ≥18 years
- Receipt of care at a Lira or Gulu clinic
- Confirmed heart failure diagnosis (ie, medical report or other supporting information)
- Ability to provide informed consent

#### Data Analysis

All interviews will be audio recorded, deidentified, and transcribed verbatim. Interviews conducted in local languages will be translated into English, and the resultant translation will be reviewed by a local staff member from each site to confirm its accuracy. Interview transcripts will be analyzed using conventional content analysis and the Indigenous method of *2-eyed seeing* developed by Mi’kmaq Elder Albert Marshall [32]. The process of *2-eyed seeing* is defined as a metaphor for negotiating between 2 cultures, where one eye is grounded in Indigenous (in this case, Ugandan) knowledge, whereas the other eye is grounded in Westernized knowledge to allow multiple perspectives to be better recognized for their own value [32]. To support the application of this method, all transcripts will be analyzed by 1 member of the UHN team and 1 member of the local clinic team, where each researcher will also use the memoing technique to document their reflective notes [33]. Reflective notes will then be shared between the UHN and local clinic team member during each analytic discussion meeting to optimize the sharing of diverse worldviews. Each team member will be given the opportunity to reflect on their agreement with or disapproval of the interpreted findings. Discrepancies regarding data interpretation will be shared with the local clinic team lead for further contextual analysis. Following this, both researchers will use the “value-adding” approach by Eakin and Gladstone [34] to further explore the analytic interpretation of the thematic findings in relation to the survey data [30].

### Phase 3: Design and System Adaptation

Using both the qualitative and quantitative data from phase 2, a set of system design requirements will be codeveloped with each site to guide the community-specific adaptation of the Medly Uganda program. In accordance with the principles of UCD and community-based research, system adaptations will take place over a series of iterations to allow for feedback on and refinement to the prototype [22,35]. The study team will set weekly co-design meetings via Zoom (Zoom Video Communications) with each site to review the summarized data from phase 2, as well as to discuss the findings in relation to the proposed system adaptations. This study recognizes that stakeholders may prefer to provide feedback through other modes of engagement, such as focus groups, community-based workshops, or individual interviews. Thus, using the stakeholder guidance from phase 1, the study team will work collaboratively with the lead nurses at each site to establish the appropriate mode to conduct the co-design meetings and incorporate community feedback. At the end of each meeting, stakeholders will be asked to complete the I-RREACH tool’s participant evaluation survey to ensure that their feedback has been suitably obtained.

This phase will also integrate the method of *2-eyed seeing* for the development of the system design requirements [32]. Specifically, during the first co-design meeting, all stakeholders will be given the opportunity to engage in a leadership role to summarize the core research findings into a series of program features. These design features will be reviewed at the subsequent stakeholder co-design meetings for discussion. This study aims to have at least one representative from the community be involved in reviewing each iteration alongside the UHN team member. Co-design meetings will be held until a mutual agreement regarding the program design requirements is reached between stakeholders.

### Phase 4: System Validation and Evaluation

#### Study Procedures

Following the approval of the program adaptations, a convenience sample of patients, clinicians, and VHTs will be invited to participate in a community-based field study to evaluate the system changes. Similar to phase 2, we aim to recruit a total of 15 patients, 10 clinicians, and 5 VHTs to participate from each of the clinics in Lira and Gulu. Using the principles from systems design and community-based research, a combined cognitive walk-through and human-technology ladder–informed interview will be conducted to evaluate both the usability and cultural relevance of the adapted program [3,36,37]. The human-technology ladder provides a framework to allow for a better understanding of how individuals engage with technology across physical, psychological, team, organizational, and political dimensions. This can include hand-eye coordination, memory, team interactions, workflows, or societal implications. Through the cognitive walk-through, participants will be asked to complete a series of task scenarios (ie, symptom reporting, medication use, nurse support, and community outreach) on a provided mobile phone. The actions and responses to the system tasks will be reviewed by the study team to allow for a goal-focused evaluation of the Medly Uganda system. All task scenarios will be codeveloped with the community and clinic stakeholders. Following the cognitive walk-through, a semistructured interview will be conducted to allow for a multilayered evaluation of the program’s utility, usability, and applicability. The 5 levels of the human-technology ladder will be used within the interview guide as this framework recognizes the importance of understanding how various human factors influence individual health behaviors [38-40]. At the end of the interview, participants will be asked to complete the System Usability Scale and the I-RREACH participant evaluation survey to evaluate the usability of the system and the appropriateness of the session.

#### Recruitment

All participants from the previous phases will be invited for the system validation and evaluation. Similar to phase 2, the study team will work in partnership with each community’s stakeholders to initiate the recruitment strategy (eg, posters, community outreach, and clinic or self-referral). Clinicians and VHTs will be invited for participation through both verbal and written (ie, email and posters) invitations. Individuals who express interest in joining the study will be connected with a member of the study team through a phone call. A member of the study team will then provide the clinician or VHT with an overview regarding the study procedures and will seek to schedule a time for an in-person session. Using the guidance from the clinic and community stakeholders, we will recruit patients through clinician and VHT referrals and community outreach (ie, posters and a radio talk show). Eligible patients interested in the study will be connected with a member of the study team to review the study procedures and subsequently schedule a session date. Once written informed consent is obtained from the participants, each session will be held in a private room at the clinic. This study phase will use the same eligibility criteria as those for phase 2 regarding participant recruitment.

#### Data Analysis

All deidentified audio recordings will be transcribed verbatim for thematic analysis. Interviews conducted in the local languages will be translated into English, and the resultant translation will be reviewed by a local staff member from each site to confirm its accuracy. Similar to phase 2, each transcript will be analyzed by 1 member of the UHN team and 1 member of the local clinic team using conventional content analysis and the Indigenous method of *2-eyed seeing*. Both reviewers will also use the memoing technique to document their notes in reflection of the research aim and the guiding principles from the human-technology ladder. Quantitative analysis of the completed surveys will be conducted concurrently with the qualitative analysis to evaluate the system’s usability. All clinic and community stakeholders will also be invited to participate in the data analysis stage to provide feedback regarding whether any further system adaptations are needed. Participants interested in analyzing or receiving the core study findings were provided with a separate form to outline their contact information for the distribution of the study findings. Given the importance of equitable and transparent principles of knowledge ownership, the control and possession of all study records will be determined by the guidance from the local team. The UHN team aims to continue their collaborative engagement with the local team to appropriately distribute the study findings beyond academic platforms. This may include avenues such as radio shows, posters, or community events.

### Ethical Considerations

This study was approved by the Makerere University School of Medicine Research and Ethics Committee (Mak-SOMREC-2021-63) and the UHN Research Ethics Board (20-6022). Written informed consent will be obtained from all participants. All information obtained during the study will be held in strict confidence. To safeguard the confidentiality and security of the identifiable study data, all participants will be identified with a unique study number, and audio recordings will be erased after transcription. At the conclusion of data analysis, all unique identifiers will be destroyed. Patient participants will receive time compensation of UGX 10,000-15,000 (US $3-$4) as well as a transport refund (if applicable) for approximately UGX 10,000-15,000 (US $3-$4). Clinician participants will receive a transport refund (if applicable) and time compensation of UGX 30,000-40,000 (US $9-$12). This research complies with all the relevant national regulations and institutional policies within the study region.

## Results

The process of partnership building was initiated in June 2019, and the first stage of data collection (phase 2) began in January 2021. At the time of manuscript submission, phases 1 to 3 have been completed. Phase 4 data analysis is ongoing and expected to be completed in July 2025.

## Discussion

### Principal Findings

Many low-income populations are often labeled as vulnerable or marginalized without recognizing the system and societal constructs that have limited their ability for growth and well-being. In Uganda, the disparities between rural and urban communities represent a clear example of how the inequitable delivery of health services has impacted population health outcomes [11,12]. To effectively facilitate meaningful change, there is a need for health services to recognize the unique circumstances, challenges, and experienced barriers that affect an individual’s well-being. Many studies have begun to implement community-based research strategies to better understand the complexities of different population contexts, but they have failed to recognize the importance of putting context into perspective [1,3,12]. Despite obtaining community input, the implication of solely using traditional Western research methods involves the assumption that each individual is willing to tell their story in the same manner with the same worldview [4,32]. Unlike Western research approaches, Indigenous methodologies integrate diverse ideologies, such as “your truth is not my truth,” to emphasize the importance of learning through lived experiences and understanding the existence of different realities [41]. As part of this framework, researchers are encouraged to engage in a continuous process of self-reflection to better recognize their individual preconceptions regarding different contexts [9,11]. These approaches to research can be integrated within any population group to better understand the dynamics of different worldviews and the importance of community or family relationships; however, current research has yet to apply these methods within non-Indigenous settings.

With the burden of HF continuing to grow in Uganda and the need to understand the contextual challenges faced by remote communities, this study aims to use both Western and Indigenous methods to adapt a digital health program for the northern communities in Uganda. To our knowledge, this is one of the few studies applying Indigenous ideologies for the collaborative co-design of a digital health program in a non-Indigenous setting [5,22]. Given the number of digital health studies that have failed to progress beyond the pilot stage in Uganda, we believe that using a diverse range of community-based methods will help ensure that the contextual significance of each community’s challenges is reflected in the program’s design [12]. Specifically, by placing local voices at the center of the research and using collaborative methods to better understand the underlying contributors to various health inequities, a more comprehensive assessment of community heart health and well-being can be conducted.

### Limitations

Despite this project’s focus on community outreach in remote settings, the participants recruited were already patients of either the Gulu Regional Referral Hospital or the Lira Regional Referral Hospital. Due to the restrictions associated with the COVID-19 pandemic, limited outreach to the surrounding lower regional health centers and community groups was available. The UHN lead (SW) was unable to travel to Uganda for phases 1 and 2 due to the pandemic, leading to the bulk of the data collection being conducted by the nurse leads at each site (JA and JK), with SW attending through internet-based Zoom platform. SW was unable to fully explore the contextual insights described in phase 2 but was able to travel to facilitate the sessions in person for phases 3 and 4.

### Conclusions and Future Work

This study recognizes that, within the field of digital health, advancements in technology move at rapid speed, whereas factors related to the digital divide continue to grow [19]. With the current mismatch between technological innovation and human social structures, by integrating a community-based approach within the UCD process, it is anticipated that various cultural and clinical factors will be identified for the adaptation of the program. As the incidence of rural poverty continues to grow in Uganda, this research believes that integrating the community’s local knowledge and lived experience into the design of the Medly Uganda program will lead to the development of a meaningful intervention to improve HF outcomes. This study recognizes that the core program adaptations will be informed by feedback from Lira and Gulu that may not reflect the contextual challenges of other communities across the country. Thus, future work will seek to collaborate with other regional referral hospitals to explore the synergies and changes needed to adapt and integrate the program within existing health system workflows. By combining the intentional use of CBPR and Indigenous methods, this study will offer a replicable blueprint for the development of more meaningful global health interventions.

## Abbreviations

CBPR: community-based participatory research
HF: heart failure
I-RREACH: Intervention and Research Readiness Engagement and Assessment of Community Health Care
UCD: user-centered design
UHI: Uganda Heart Institute
UHN: University Health Network
VHT: village health team

## Data Availability

The datasets generated or analyzed during this study are available from the corresponding author on reasonable request. A results paper will also be published in follow-up to this protocol.
